# A new era for understanding and managing common pruritic skin conditions: How to define, diagnose, and treat?

**DOI:** 10.1111/1346-8138.17574

**Published:** 2024-12-12

**Authors:** Takahiro Satoh

**Affiliations:** ^1^ Department of Dermatology National Defense Medical College Tokorozawa Japan

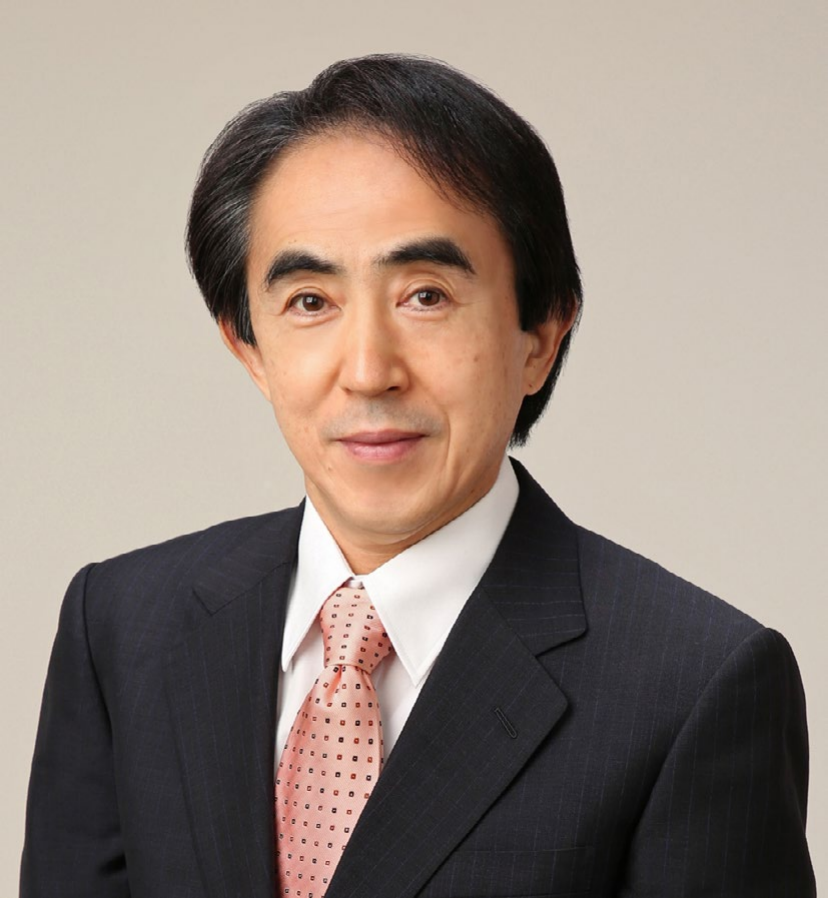

The treatment of inflammatory skin diseases is entering a new era. The advent of biologics and small‐molecule agents has facilitated the therapy of some of the previously difficult‐to‐treat skin conditions. However, there are still several pruritic skin conditions that many dermatologists find difficult to manage. Understanding the precise clinical features and actual pathological mechanisms of these diseases is essential for the development and appropriate use of new therapies. This special issue of the *Journal of Dermatology* focuses on three common pruritic skin diseases.

Eczema is the most common and well‐known pruritic disease in dermatology. However, how many dermatologists fully understand the exact nature of eczema and its characteristics? In this special issue, Tokura et al. discuss the nature of eczema. They explain the etymology and classic concept of eczema, including the ‘eczema triangle’. They also discuss the pathological mechanisms of eczema based on the current understanding of cutaneous immunology.

While we have recently gained powerful therapeutic tools for treating chronic inflammatory skin conditions, such as atopic dermatitis and psoriasis, erythroderma in the elderly remains difficult to treat. In general, the diagnosis of erythroderma is relatively straightforward. Nevertheless, despite careful examinations, it is often difficult to determine the actual cause of the disease. Yamamoto explains the concept, clinical characteristics, and pathogenesis of erythroderma in the elderly, and the difference between elderly erythroderma and elderly atopic dermatitis.

Research into itching has lagged far behind that into pain, and, for a long time, antihistamines have dominated the treatment of itching associated with skin diseases. However, recent advances in the study of itch have provided us with a wealth of knowledge about nonhistaminergic itch. In this issue, Hashimoto et al. describe the latest findings on the pathophysiology of itch. They also explain the mechanisms of generalized and localized pruritus without rash and how we can manage these conditions.

The review articles in this issue will undoubtfully help dermatologists to better understand, diagnose, and manage these common pruritic skin conditions.

